# Value of reduced glomerular filtration rate assessment with cardiometabolic index: insights from a population-based Chinese cohort

**DOI:** 10.1186/s12882-018-1098-8

**Published:** 2018-10-25

**Authors:** Hao-Yu Wang, Wen-Rui Shi, Xin Yi, Shu-Ze Wang, Si-Yuan Luan, Ying-Xian Sun

**Affiliations:** 1 Department of Cardiology, The First Hospital of China Medical University, 155 Nanjing North Street, Heping District, Shenyang, 110001 China; 2 Department of Cardiovascular Medicine, Beijing Moslem Hospital, Beijing, 100054 China; 3 Department of Computational Medicine and Bioinformatics, University of Michigan, 100 Washtenaw Avenue, Ann Arbor, MI 48109 USA; 4 West China School of Medicine, Sichuan University, #37 Guoxue Alley, Chengdu, 610041 China

**Keywords:** Cardiometabolic index, Dyslipidemia, Obesity, Reduced eGFR, Sex-specific, Visceral adipose tissue

## Abstract

**Background:**

Recent studies have suggested that cardiometabolic index (CMI), a novel estimate of visceral adipose tissue, could be of use in the evaluation of cardiovascular risk factors. However, the potential utility and clinical significance of CMI in the detection of reduced estimated glomerular filtration rate (eGFR) remains uncertain. The purpose of this study was to investigate the usefulness of CMI in assessing reduced eGFR in the general Chinese population.

**Methods:**

This cross-sectional analysis included 11,578 participants (mean age: 53.8 years, 53.7% females) from Northeast China Rural Cardiovascular Health Study (NCRCHS) of general Chinese population (data collected from January 2013 to August 2013). CMI was calculated by triglyceride to high density lipoprotein cholesterol ratio multiply waist-to-height ratio. Reduced eGFR was defined as eGFR< 60 ml/min per 1.73m^2^. Multivariate regressions were performed to determine CMI’s association with eGFR value and eGFR reduction, ROC analyses were employed to investigate CMI’s discriminating ability for decreased eGFR.

**Results:**

The prevalence of reduced eGFR was 1.7% in males and 2.5% in females. CMI was notably more adverse in reduced eGFR groups, regardless of genders. In fully adjusted multivariate linear models, each 1 SD increment of CMI caused 3.150 ml/min per 1.73m^2^ and 2.411 ml/min per 1.73m^2^ loss of eGFR before CMI reached 1.210 and 1.520 in males and females, respectively. In logistic regression analyses, per 1 SD increase of CMI brought 51.6% additional risk of reduced eGFR in males while caused 1.347 times of risk in females. After divided into quartiles, people in the top quartile of CMI had higher adjusted ORs of having reduced eGFR, with ORs of 4.227 (1.681, 10.627) and 3.442 (1.685–7.031) for males and females respectively. AUC of CMI was revealed to be 0.633 (0.620–0.646) in males and 0.684 (0.672–0.695) in females.

**Conclusions:**

Higher CMI was independently associated with greater burden of reduced eGFR, highlighting VAT distribution and dysfunction as a potential mechanism underlying the association of obesity with kidney damage and adverse cardiovascular outcomes. The findings from this study provided important insights regarding the potential usefulness and clinical relevance of CMI in the detection of reduced eGFR among general Chinese population.

## Background

Chronic kidney disease (CKD) was a disease with heterogeneous etiology that caused 15.8 deaths per 100,000 people worldwide in 2013 and was estimated to attack 119.5 million Chinese people in the year of 2012 [1, 2]. Besides, CKD has been identified to be related with multiple cardiovascular risk factors like hypertension and diabetes mellitus (DM), revealing its close association with cardiovascular health [3, 4]. Therefore, CKD has brought great burden to the health-care work and economy [5, 6]. Knowing the poor outcomes and high cost that caused by CKD as a worldwide public health problem, it is a good choice to focus on the early detection of reduced estimated glomerular filtration rate (eGFR) for avoiding the progression of CKD. Accordingly, early detection of reduced eGFR is critically needed for timely CKD prevention and overall improvement in prognosis.

There is an wealthy evidence that dyslipidemia and obesity are strongly related to the deterioration of renal function, even in the early stage [7, 8]. Dyslipidemia in CKD often exhibits a pattern of increased triglyceride (TG) levels and decreased high density lipoprotein cholesterol (HDL-C) levels [7]. Findings revealed that increased TG level was significantly correlated with CKD [9–11]. Furthermore, as a combination of 2 characteristics of dyslipidemia in CKD, TG to HDL-C ratio (TG / HDL-C) has also been identified to strongly associated with decline of eGFR in participants without CKD and rapid decrement of eGFR in participants with CKD [12–14]. Obesity is another condition that strongly correlated with decreased eGFR or CKD [8, 15]. As the most widely used index of obesity, body mass index (BMI) was revealed to correlated with reduced eGFR, CKD and end stage renal disease (ESRD) [16, 17]. However, BMI did not take abdominal obesity into consideration when reflecting obesity status. Therefore, some indexes such as waist circumference (WC) and waist-to-height ratio (WHtR) were designed to be measurements of central obesity [18]. And they have already showed strong associations with CKD [19–22]. Nevertheless, comparisons between BMI, WC and WHtR did not provide sufficient evidence for anyone of them to be a premier marker of CKD with great sensitivity or specificity [19–22]. Moreover, indexes of abdominal obesity were insufficient to detecting visceral adipose tissue (VAT), which was elucidated to possess a more adverse effect on CKD development than subcutaneous adipose tissue (SAT) [23, 24]. However, when cooperating with aforementioned indexes of dyslipidemia, their strength to discriminating VAT got reinforcement [24]. Taken together, combination of dyslipidemia and abdominal obesity could improve the identification of VAT and therefore refine the detecting of reduced eGFR.

Recently, a novel marker named “cardiometabolic index (CMI)” has been put forward by Ichiro Wakabayashi [25]. CMI can be considered as an ideal marker to recognize VAT because of its integration of dyslipidemia and abdominal obesity. This emerging marker has been shown in several studies to be a useful screening tool for various populations, identifying those with a deteriorated metabolic profile and higher risk for cardiovascular disease, such as left ventricular geometry abnormalities, hyperuricemia, diabetes, hypertension, and ischemic stroke [25–29]. It is unclear, however, if CMI can be an identifier of reduced eGFR, independent of cardiovascular risk factors and hypertension. Accordingly, the present study was designed to test whether or not higher CMI increase the risk of reduced eGFR in the general Chinese population.

## Methods

### Study population

This study was part of a large cross-sectional population-based epidemiological investigation that described the prevalence, incidence, and natural history of cardiovascular risk factors among 11,956 permanent residents (≥35 years of age) in rural areas of China from January 2012 to August 2013. The full details regarding the design and rationale of the study were extensively described elsewhere [26, 30, 31]. Briefly, the study adopted a multistage, stratified random cluster-sampling scheme. In the first stage, 3 counties (Dawa, Zhangwu, and Liaoyang County) were selected from the eastern, southern, and northern region of Liaoning province. In the second stage, 1 town was randomly selected from each county (a total of 3 towns). In the third stage, 8 to 10 rural villages from each town were randomly selected (a total of 26 rural villages). Participants with pregnancy, malignant tumor, or mental disorder were excluded from the present study. The study protocol complied with the Second Helsinki Declaration (and recent amendments) and was approved by the Ethics Committee of China Medical University (Shenyang, China), and all study participants provided written informed consent. Patients with missing data for any of CMI components or other variables analyzed in the study (*n* = 378) were excluded. Accordingly, the final study cohort was composed of 11,578 subjects.

### Data collection and measurements

Detailed process about data collection and measurements was described by previous publications from our research team that involved in the same survey as this study [26, 30, 31]. Cardiologists and nurses completed a training and passed a test before they were allowed to conduct the questionnaire which collected information about demographic data, health-related behavior, anthropometric parameters, dietary condition, current medicine usage condition, history of cardiovascular disease (CVD).

A carefully designed questionnaire was used to collect data from subjects. A central steering committee with a subcommittee conducted the quality control of the information collection process. Subjects were divided into two groups: Han and others. Education level was regarded as three ordinal groups: primary school or below, middle school, high school or above. Three ordinal groups (≤5000 CNY,5000–20,000 CNY,> 20,000 CNY) were utilized to represent the family annual income level. Diet score was calculated according to the collected information about vegetable and meat consumption, a lower score meant lower meat consumption and higher vegetable consumption. Detailed information about diet score has been reported in a prior study from our team [32]. Physical activity level was acquired in both work and off hours, and divided into three levels (low, middle, high). Messages of current smoking and alcohol intake status were collected through a series of questions about smoking and drinking history of participants. Medicine usage such as anti-hypertensive, anti-diabetic and lipid-lowering drugs was recorded in the questionnaire as well. History of CVD included coronary heart disease, arrhythmia, heart failure, and stroke. Meanwhile, history of kidney disease was defined as nephritis, kidney dysfunction, renal stones, renal tumor, and autoimmune kidney disease.

After subjects completed a 5 min rest in sitting position with relaxation, two randomly selected trained medical staffs performed the blood pressure measurements for them. Three consecutive readings were recorded and their mean value was taken into statistical analyses.

With regard to the measurements of anthropometric indices, subjects were requested to wear in light clothing without shoes. Standard weight was measured to the nearest 0.1 kg by using a calibrated digital scale. Subjects held in a standing position when a calibrated stadiometer was used to quantify their standard height to the nearest 0.1 cm. As for the measurement of WC, elastic measuring tapes was used to get the readings in a horizontal position at 1 cm above the umbilicus. All of the above measurements were performed twice and their mean values were used for analyses.

Detailed delineation of the process of storage and methods of laboratory measurements was reported in our previous studies [26, 30, 31]. Briefly, fasting (12 h overnight) blood samples were collected through venipuncture. And these samples were separated and frozen at − 20 °C within 1 h after the collection, and then transported to a laboratory with certification for examination. An Olympus AU 640 auto analyzer (Olympus, Kobe, Japan) was used for biochemical analyses. All laboratory equipment was calibrated and blinded duplicate samples were used.

### Definitions

BMI was calculated as mean weight divided by mean height squared (kg/m^2^). WHtR was acquired as WC divided by mean height. CMI was obtained by the following equation [25]: CMI = TG/HDL-C × WHtR. lipid accumulation product (LAP) was calculated according to the sex-specific formula [33]: LAP = TG (mmol/L) × [WC (cm)-58] for females and LAP = TG (mmol/L) × [WC (cm)-65] for males. And visceral adiposity index (VAI) was determined by using the following formula [34]: Males: VAI = [WC / 39.68 + (1.88 × BMI)] × (TG / 1.03) × (1.31 / HDL); Females: VAI = [WC / 36.58 + (1.89 × BMI)] × (TG / 0.81) × (1.52/HDL).

eGFR was calculated according to CKD-EPI equation [35]. Reduced eGFR was defined as eGFR < 60 ml/min per 1.73m^2^ [36]. Diagnostic criteria of hypertension were mean systolic blood pressure (SBP) equal to or greater than 140 mmHg and/or diastolic blood pressure (DBP) at least 90 mmHg and/or participants who were on antihypertensive medications or self-reported previous diagnosed hypertension [37]. Diagnosis of diabetes based on the American Diabetes Association criteria: fasting plasma glucose (FPG) ≥ 7.0 mmol/L and/or self-reported previous diagnosis history or receiving plasma glucose lowering therapy [38].

### Statistical analyses

Analyses were performed in a sex-specific manner. Continuous variables were expressed as mean values ± standard deviation (SD) or as median (interquartile range) if appropriate. Categorical variables were depicted as frequencies (percentages). Continuous variables were compared between groups with Student’s t test or Mann-Whitney test according to their distribution, in the meantime, categorical variables were also compared between groups by using χ^2^ test [39]. As for the comparison of ordinal categorical variables (education level, family annual income, physical activity), rank-sum test was performed in order to get full utilization of the ordinal information. Before inferential analyses, CMI values were log transformed due to highly skewed distributions. The chi-square linear-by-linear association test was used to reveal linear trends across the quartiles of CMI for percentages of prevalence of reduced eGFR. Multivariate linear regression analysis was conducted to evaluate the independent effect of CMI on eGFR value. And logarithmic likelihood ratio test was employed to compare the one-line linear regression model with a two-piecewise linear model. Furthermore, multivariate logistic regression analysis was performed to explore the isolated association of CMI as a continuous variable and as quartiles with the prevalence of reduced eGFR. Sex-specific odds ratios (ORs) for every SD change of CMI to identify the risk of reduced eGFR were acquired, the results were displayed as ORs and 95% confidence intervals (95% CI). Lastly, receiver-operating characteristic (ROC) curve was employed to investigate the optimal cut-off value of CMI to detect the presence of reduced eGFR. Area under the curve (AUC) was used to compare the discriminating ability of CMI and other indexes. All of the statistical analyses involved were performed by SPSS 25.0 software (IBM corp), statistical software packages R (http://www.R-project.org, The R Foundation) and EmpowerStats (http://www.empowerstats.com, X&Y Solutions, Inc., Boston, MA), Prism 7.0 software (Graphpad software, Inc) and MedCalc version 18.5 (MedCalc software, Belgium), a two-tailed *P* value less than 0.05 indicated statistical significant.

## Results

After excluding ineligible participants, we finally took 11,578 subjects into analyses, the results were shown in Table 1. Of these subjects, 46.3% were males. The mean age of total population was 53.8 years while people with eGFR decrement were elder than their counterparts in both genders. The prevalence of reduced eGFR was 1.7% in males while females exhibited a higher rate of 2.5%. As for the demographic information, education level, family annual income, diet score and physical activity exhibited significant lower levels in patients with reduced eGFR. Contradictorily, female patients were more likely to smoke at present but male patients tended not to be a current smoker. Only males showed significant difference on alcoholic status between groups, but the results revealed that normal eGFR subjects had greater possibility to be a current drinker. With regard to laboratory test results, FPG, serum uric acid, TG and TG/HDL-C confirmed a remarkable augmentation among eGFR decrement patients, together with a dramatically decline of HDL-C in patients. SBP, height, WC, WHtR showed great increment when comparing CKD patients with their counterparts regardless of sex. But DBP only exhibited higher level in male patients while less weight was specific to female patients. Prevalence of hypertension and diabetes were noticeable higher among subjects with reduced eGFR for both genders, and the usages of anti-hypertensive, anti-diabetic and lipid-lowering drug were more popular in reduced eGFR group. Concordantly, the prevalence of cardiovascular and kidney diseases history showed dramatic augmentation in eGFR reduction patients of both genders. Within our expectation, patients with reduced eGFR of both genders experienced pronounced increment of CMI, VAI as well as LAP.

**Table 1:** Characteristics of participants with reduced eGFR stratified by sex

Variables	Males (*n* = 5360)			Females (*n* = 6218)		
	Reduced eGFR (*n* = 90)	Normal eGFR (*n* = 5270)	*P* value*	Reduced eGFR (*n* = 155)	Normal eGFR (*n* = 6063)	*P* value*
Age (years)	68.78 ± 10.41	54.11 ± 10.64	< .001	68.69 ± 8.87	52.99 ± 10.08	< .001
Race (Han) (%)	87 (96.7)	4987 (94.6)	0.394	152 (98.1)	5747 (94.8)	0.068
Education level (%)			< .001			< .001
Primary school or below	57 (63.3)	2177 (41.3)		135 (87.1)	3398 (56.0)	
Middle school	26 (28.9)	2487 (47.2)		18 (11.6)	2187 (36.1)	
High school or above	7 (7.8)	606 (11.5)		2 (1.3)	478 (7.9)	
Income level (CNY) (%)			< .001			< .001
≤ 5000	28 (31.1)	693 (13.1)		41 (26.5)	680 (11.2)	
5000 – 20,000	45 (50.0)	2827 (53.6)		77 (49.7)	3361 (55.4)	
> 20,000	17 (18.9)	1750 (33.2)		37 (23.9)	2022 (33.3)	
Diet score	2.24 ± 0.96	2.55 ± 1.10	0.008	1.68 ± 1.03	2.15 ± 1.11	< .001
Physical activity (%)			< .001			< .001
Low	52 (57.8)	1158 (22.0)		100 (64.5)	2133 (35.2)	
Middle	36 (40.0)	3817 (72.4)		42 (27.1)	3587 (59.2)	
High	2 (2.2)	295 (5.6)		13 (8.4)	343 (5.7)	
Current smoking (%)	28 (31.1)	3030 (57.5)	< .001	43 (27.7)	987 (16.3)	< .001
Current alcohol intake (%)	14 (15.6)	2421 (45.9)	< .001	3 (1.9)	180 (3)	0.452
FPG (mmol/L)	5.74 (5.35 – 6.67)	5.60 (5.22 – 6.09)	0.010	5.78(5.30 – 7.05)	5.49 (5.12 – 5.99)	< .001
Serum uric acid (μmol/L)	447.52 ± 122.56	331.80 ± 81.34	< .001	363.21 ± 94.01	253.06 ± 64.66	< .001
TG (mmol/L)	1.41 (1.10 – 2.07)	1.22 (0.86 – 1.88)	0.006	1.86 (1.30 – 2.55)	1.24 (0.89 – 1.88)	< .001
HDL-C (mmol/L)	1.21 ± 0.27	1.41 ± 0.42	< .001	1.35 ± 0.41	1.41 ± 0.34	0.018
TG/HDL-C	1.19 (0.80 – 2.11)	0.91 (0.57 – 1.57)	< .001	1.42 (0.92 – 2.24)	0.92 (0.59 – 1.51)	< .001
SBP (mmHg)	161.58 ± 26.32	143.36 ± 22.43	< .001	154.16 ± 26.14	139.79 ± 23.87	< .001
DBP (mmHg)	89.01 ± 16.20	83.69 ± 11.71	0.003	82.57 ± 14.77	80.52 ± 11.41	0.088
Height (cm)	164.08 ± 5.56	166.46 ± 6.35	< .001	152.91 ± 5.91	155.69 ± 6.06	< .001
Weight (kg)	68.52 ± 12.06	68.61 ± 11.10	0.940	58.08 ± 10.64	60.33 ± 10.11	0.006
BMI (kg/m^2^)	25.36 ± 3.65	24.72 ± 3.54	0.091	24.77 ± 3.95	24.86 ± 3.76	0.778
WC (cm)	87.05 ± 10.36	83.74 ± 9.75	0.003	84.07 ± 10.37	81.20 ± 9.71	< .001
WHtR	0.53 ± 0.06	0.50 ± 0.06	< .001	0.55 ± 0.07	0.52 ± 0.06	< .001
hypertension (%)	74 (82.2)	2818 (53.5)	< .001	120 (77.4)	2907 (47.9)	< .001
diabetes (%)	22 (24.4)	508 (9.6)	< .001	41 (26.5)	629 (10.4)	< .001
Anti-hypertensive drug (%)	50 (55.6)	641 (12.2)	< .001	73 (47.1)	989 (16.3)	< .001
Anti-diabetic drug (%)	13 (14.4)	148 (2.5)	< .001	20 (12.9)	278 (4.6)	< .001
Lipid-lowering drug (%)	7 (7.8)	159 (3.0)	0.021	12 (7.7)	202 (3.3)	0.003
history of CVD (%)	46 (51.1)	781 (14.8)	< .001	62 (40.0)	1092 (18.0)	< .001
history of kidney disease	5 (5.6)	60 (1.1)	< .001	11 (7.1)	77 (1.3)	< .001
eGFR (ml/min per 1.73m^2^)	54.81 (46.46 – 57.67)	95.44 (87.00 – 103.44)	< .001	52.90 (46.89 – 57.47)	93.23 (82.89 – 103.29)	< .001
LAP (cm·mmol/L)	31.88 (18.63 – 51.35)	21.79 (10.62 – 43.35)	0.001	44.94 (26.00 – 77.56)	27.84 (16.00 – 50.25)	< .001
VAI	1.51 (1.00 – 2.60)	1.12 (0.68 – 1.98)	< .001	2.68 (1.78 – 4.17)	1.67 (1.06 – 2.78)	< .001
CMI	0.62 (0.41 – 1.10)	0.45 (0.27 – 0.82)	< .001	0.81 (0.49 – 1.26)	0.47 (0.29 – 0.81)	< .001

Data are expressed as mean ± standard deviation(SD) or median (interquartile range) and numbers (percentage) as appropriate. Income level: family annual income; *CNY* Chinese currency (1CNY = 0.15 USD), *SBP* systolic blood pressure, *DBP* diastolic blood pressure, *FPG* fasting plasma glucose, *CVD* cardiovascular disease, includes coronary heart disease, arrhythmia, heart failure, and stroke; Kidney disease: includes nephritis, kidney dysfunction, renal stones, renal tumor, and autoimmune kidney disease. Body mass index; *WC* waist circumference, *WHtR* waist-to-height ratio, *TG* triglyceride, *HDL-C* high density lipoprotein cholesterol, *TG/HDL-C* triglyceride to high density lipoprotein cholesterol ratio, *eGFR* estimated glomerular infiltration rate, *LAP* lipid accumulation product, *VAI* visceral adiposity index, *CMI* cardiometabolic index

*Comparisons of category variables between groups were tested by chi-square test or rank-sum test (ordinal category variables) and comparisons for continuous variables between groups were tested by Student’s t or Mann-Whitney test

**Fig. 1:**
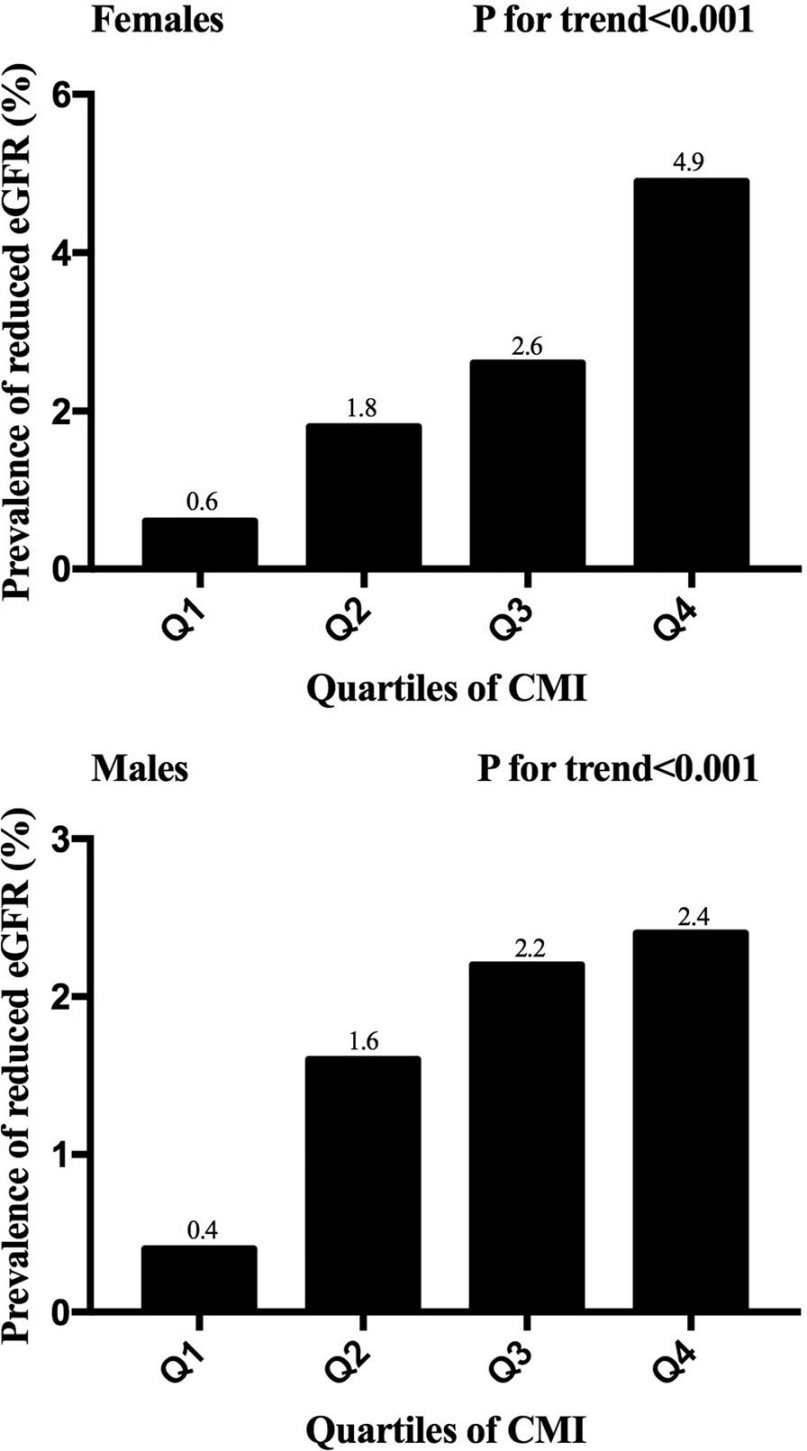
The prevalence of reduced eGFR by quartiles of CMI. Prevalent decreased eGFR increased proportionally across ascending quartiles of CMI in both genders (P for trend< 0.05). Abbreviations: CMI, cardiometabolic index; eGFR: estimated glomerular filtration rate

Quartile analyses revealed gradient correlation between CMI and prevalence of reduced eGFR, as displayed in Fig. 1. When comparing the top quartile with the bottom one, males exhibited a 6.0-fold change for the probability of eGFR reduction while females showed a higher fold change of 8.2. Both genders confirmed linear trends of prevalent eGFR reduction across quartiles of CMI (all P for trend< 0.001).

Results of linear regression analyses identified robust negative association between CMI with eGFR. As presented in Table 2, logarithmic likelihood ratio test identified the association between CMI and eGFR was non-linear in both genders after adjustment of age, race, education level, family annual income, diet score, physical activity, current smoking, alcohol intake, hypertension, diabetes, antihypertensive drug, antidiabetic drug, lipid-lowering drug, history of cardiovascular and kidney diseases. In the full model of males, the two-piecewise linear model revealed a rapid decrease of eGFR value along with the early increase of normalized CMI, with a β value of − 3.150 (− 3.589, − 2.712). However, when normalized CMI reached 1.113 (equaled to 1.210 of CMI), the change of eGFR shifted to a mild increase, with a β value of 1.906 (0.675, 3.138). Similarly, in the complete model of females, the eGFR also suffered a loss of 2.411 ml/min per 1.73m^2^ for each SD increase of CMI before normalized CMI arrived to 1.472 (equaled to 1.520 of CMI). But after that, the eGFR began to have a increment of 2.268 ml/min per 1.73m^2^ along with every single unit increase of normalized CMI.

**Table 2:** Evaluation of the impact of CMI on eGFR value with the use of piecewise linear regression^a^

		Unadjusted	MV adjusted
Males			
Linear model	β Value (95% CI) *P* value	−1.850 (−2.259, −1.440) < 0.001	−2.214 (−2.561, −1.866) < 0.001
Non-linear model	Breakpoint (K)	1.024	1.113
	β 1 (<K) (95% CI) *P* value	−3.324 (−3.863, − 2.786) < 0.001	−3.150 (−3.589, − 2.712) < 0.001
	β 2 (>K) (95% CI) *P* value	3.777 (2.371, 5.182) < 0.001	1.906 (0.675, 3.138) 0.002
	Logarithmic likelihood ratio test *P* value	< 0.001	< 0.001
Females			
Linear model	β Value (95% CI) *P* value	−4.046 (−4.438, −3.654) < 0.001	−1.914 (−2.262, − 1.566) < 0.001
Non-linear model	Breakpoint (K)	1.34	1.472
	β 1 (<K) (95% CI) *P* value	−5.001 (−5.472, −4.530) < 0.001	−2.411 (− 2.813, − 2.010) < 0.001
	β 2 (>K) (95% CI) *P* value	2.478 (0.636, 4.320) 0.008	2.268 (0.547, 3.988) 0.010
	Logarithmic likelihood ratio test *P* value	< 0.001	< 0.001

Abbreviations: *CMI* cardiometabolic index, *OR* odds ratio, *95% CI* 95% confidence interval. Linear model: model that presumes the association between CMI and eGFR is linear. Non-linear model: model that presumes the association between CMI and eGFR is non-linear and has breakpoint. Unadjusted: no adjustment; MV adjusted: multivariable adjusted model, includes age, race, education level, family annual income, physical activity, current smoking, current alcohol intake, hypertension, diabetes, antihypertensive drug, antidiabetic drug, lipid-lowering drug, history of cardiovascular disease and kidney disease. ^a^Two-step linear regression model was applied to explore the non-linear association between CMI and eGFR

Logistic regression analyses confirmed the intensive relationship between CMI with reduced eGFR, the results were arranged in Table 3. In males, per 1 SD increase of CMI still caused 52% of additional risk for eGFR reduction after adjusting all included cofounders. When divided into quartiles, highest quartile of CMI displayed 4.2 times risk for developing eGFR decrement compared with lowest category in the full model, and there was a significant linear trend for the risk of eGFR reduction across the quartiles of CMI (P for trend< 0.001). Similar results were observed in female participants, risk of eGFR reduction got an elevation of 34.7% for every single SD increment of CMI. Furthermore, the ORs for top quartile compared with bottom group was 3.442 (1.685–7.031), and the linear trend across quartiles also existed (*P* for trend< 0.001). We further conducted a test for the interaction between genders and CMI, and the results displayed to be insignificant, identifying the robust association between CMI and reduced eGFR across genders.

**Table 3:** Sex-specific logistic regression models for reduced eGFR with CMI

	Males				Females				*P* for interaction
	Unadjusted OR (95% CI)	*P* value	MV adjusted OR (95% CI)	*P* value	Unadjusted OR (95% CI)	*P* value	MV adjusted OR (95% CI)	*P* value	
CMI (Per 1 SD increase)	1.467 (1.211, 1.777)	< 0.001	1.516 (1.182, 1.943)	0.001	1.760 (1.519, 2.040)	< 0.001	1.347 (1.122, 1.618)	0.001	0.172
Quartiles of CMI									
Quartile 1	1.000		1.000		1.000		1.000		
Quartile 2	3.711 (1.500, 9.182)	0.005	2.717 (1.061, 6.957)	0.037	2.835 (1.372, 5.856)	0.005	2.285 (1.069, 4.888)	0.033	
Quartile 3	5.092 (2.112, 12.273)	< 0.001	3.867 (1.551, 9.640)	0.004	4.187 (2.090, 8.387)	< 0.001	2.509 (1.204, 5.225)	0.014	
Quartile 4	5.439 (2.267, 13.052)	< 0.001	4.227 (1.681, 10.627)	0.002	7.939 (4.091, 15.408)	< 0.001	3.442 (1.685, 7.031)	< 0.001	
*P* value for trend		< 0.001		0.001		< 0.001		< 0.001	

*MV* indicates multivariable, *OR* odds ratio, *CI* confidence interval, *CMI* cardiometabolic index. Cut points for CMI: Men: ≤ 0.27, > 0.27 and ≤ 0.46, > 0.46 and ≤ 0.83, > 0.83; Women: ≤ 0.30, > 0.30 and ≤ 0.48, > 0.48 and ≤ 0.82, > 0.82. MV model adjusted for age, race, education level, family annual income, diet score, physical activity, current smoking, current alcohol intake, hypertension, diabetes, antihypertensive drug, antidiabetic drug, lipid-lowering drug, history of cardiovascular disease (coronary heart disease, arrhythmia, heart failure, and stroke) and kidney disease (nephritis, kidney dysfunction, renal stones, renal tumor, and autoimmune kidney disease)

ROC analyses showed a significant AUC value of CMI for discriminating eGFR reduction, the results were summarized in Table 4. In males, our findings displayed that CMI possessed the greatest AUC (AUC: 0.633, 95% CI: 0.620–0.646) among various indexes, statistically significantly higher than that of LAP (AUC 0.633 vs. 0.606, *P* = 0.036) and BMI (AUC 0.633 vs. 0.544, *P* = 0.004), and there was also a trend for CMI to be superior than VAI (0.627, 0.614–0.640). Meanwhile, CMI showed a sensitivity of 88.9% and a specificity of 37.1%. In females, CMI had an AUC (AUC: 0.684, 95% CI: 0.672–0.695) which was comparable to that of VAI (AUC: 0.688, 95% CI: 0.676–0.699, *P* value for difference = 0.148) and statistically significantly greater than that of LAP (AUC 0.684 vs 0.660, *P* = 0.047), WC (AUC 0.684 vs 0.588, *P* < 0.001) and BMI (AUC 0.684 vs 0.500, *P* < 0.001). Furthermore, CMI exhibited the greatest sensitivity (89.0%) in this gender. However, the specificity was still low and was given to 39.8%.

**Table 4:** AUC for indexes to discriminate eGFR reduction in females and males

Variables	AUC (95%CI)	*P* value	Cut-off according to Youden’s index	Sensitivity (%)	Specificity (%)
Males					
CMI	0.633 (0.620–0.646)^a,c^	< 0.001	> 0.35	88.9%	37.1%
VAI	0.627 (0.614–0.640)^c^	< 0.001	> 0.83	90.0%	34.7%
LAP	0.606 (0.593–0.619)^c^	< 0.001	> 18.33	77.8%	43.6%
WC	0.589 (0.576–0.602)^c^	0.001	> 79.1	83.3%	35.0%
BMI	0.544 (0.531–0.558)	0.135	> 22.02	88.9%	22.8%
Females					
CMI	0.684 (0.672–0.695)^a,b,c^	< 0.001	> 0.39	89.0%	39.8%
VAI	0.688 (0.676–0.699)^a,b,c^	< 0.001	> 1.69	80.0%	50.4%
LAP	0.660 (0.648–0.672)^b,c^	< 0.001	> 36.12	65.2%	61.8%
WC	0.588 (0.576–0.600)^c^	< 0.001	> 84.20	52.9%	64.0%
BMI	0.500 (0.488–0.513)	0.995	≤ 27.81	76.1%	20.1%

Abbreviations: *AUC* area under the ROC curve, *95% CI* 95% confidence interval, *CMI* cardiometabolic index, *VAI* visceral adiposity index, *LAP* lipid accumulation product, *WC* waist circumference, *BMI* body mass index

^a^indicates a significant larger as compared to LAP;

^b^indicates a significant larger as compared to WC;

^c^indicates a significant larger as compared to BMI

## Discussion

Our study showed that in this large, community-based cohort of middle-aged Chinese individuals, CMI, a novel measure of VAT, was independently and robustly associated with the presence of reduced eGFR in both genders. CMI could reflect not only VAT but also pathologic process that resulted in impaired kidney function, thus provided a clinical clue for related basic researches. As our results revealed the potential of CMI as a screening marker of reduced eGFR, further understanding of the underlying pathophysiology should improve strategies to prevent and potentially reverse detrimental kidney failure and cardiovascular outcomes, especially in people with poor socioeconomic conditions.

Dyslipidemia is a condition that always appears in the development of CKD, even in the early stage of eGFR reduction, and the major components of dyslipidemia in CKD has been revealed to be increased TG level and decreased HDL-C level [7]. Indexes that represent this specific pattern has been identified to have a strong correlation with CKD. For instance, Hou et al. revealed that TG level was closely associated with mildly decreased eGFR even among middle aged or elderly Chinese population. It was worthy to note that this relationship existed when TG level was still in the normal range. This finding gave us an implication that increased TG level could appear in the early process of decreased eGFR [9]. Lee et al. identified hypertriglyceridemia as an independent risk factor of progressed CKD, confirmed the association persistent in middle and late stage of CKD [11]. Furthermore, Tozawa et al. and Shimizu et al. provided compelling evidences that TG had an independent effect to increase the risk of eGFR decrement from longitudinal studies, Tozawa et al. also identified HDL-C positively related to eGFR value and revealed TG had an impact on the development of proteinuria [10, 40]. Apart from above investigations, researches also identified the utility of TG/HDL-C in identifying eGFR reduction as it incorporated both TG and HDL-C level. Ho et al. demonstrated the correlation between TG/HDL-C with CKD, and Wen et al. even showed the advantage of TG/HDL-C when compared with TG alone with respect to the development of CKD, confirmed our aforementioned hypothesis [13, 14]. However, there was one point that worth to be mentioned, although TG/HDL-C had a robust association with CKD, it was not an ideal marker of eGFR reduction due to its low sensitivity and specificity [13].

As another condition that always accompany with CKD, obesity is related to multiple risk factors of CKD, such as hypertension and diabetes [41, 42]. Its direct association with CKD has been evaluated through indexes. As the most widely used index of obesity, BMI has already been elucidated to have a connection with new-onset CKD, CKD progression and end stage renal failure [16, 17, 43]. However, since BMI do not consider adipose tissue distribution, there is no doubt that BMI is unsuitable for reflecting the relationship between obesity phenotype with CKD. Therefore, indexes of abdominal obesity such as WC and WHtR have been widely investigated for their associations with CKD. Among them, WHtR was found to have greatest application potential for detecting CKD by several studies [19, 20]. Theoretically, by using height to standardize WC value, WHtR was design to be a superior reflection of abdominal obesity since people with different height were supposed to have different standard WC value. Nevertheless, studies did not support any of these indexes to be an eligible marker with great sensitivity or specificity for detection of reduced eGFR [19–22]. Meanwhile, studies found that VAT contributed more to CKD than SAT [23]. Indexes like WC hadd no power to distinguish VAT from SAT alone but could recognize VAT correctly when working together with TG [24, 44]. Taking all above messages together, we can speculate that indexes contain information about dyslipidemia and abdominal obesity should do better in the identification of VAI and be able to refine the recognition of eGFR reduction.

Indexes that integrate both abdominal obesity and dyslipidemia has been proposed. Determined by WC and TG levels, LAP was put forward for recognizing cardiovascular risk [33], and it has also been identified to possess a stronger association with CKD than BMI, WC and WHtR [45]. Later, a concept named VAI was posited as an indicator of cardiometabolic risk with an additional consideration of HDL-C and BMI when compared with LAP [34]. In recent studies, VAI showed strong association with CKD and greater potential as a marker of eGFR reduction than LAP as well as traditional anthropometric indexes [45, 46]. However, these two novel indexes still have limitations. Both LAP and VAI have sex-specific equations, and VAI has a sophisticated algorithm, these will increase the complexity of calculation. As for LAP, information about HDL-C is not utilized, which is an important aspect of dyslipidemia in CKD. Moreover, height info is also wasted by LAP, but people with different height should have different WC standard. Last but not least, although VAI possessed a greater discriminative power than LAP and other indexes, it still failed to reach satisfying sensitivity and specificity values, thus its value for becoming a marker of eGFR reduction is limited [46].

CMI is a young index which was posited by Ichiro Wakabayashi in 2015 [25]. Different from LAP and VAI, it reflects both characteristics of dyslipidemia in CKD and accurate central obesity status through a simple, generalized eq. CMI has also been identified to correlate with multiple cardiovascular risk factors, implicated to have a great potential in screening related diseases [25–29]. Since many of these diseases has been identified to be risk factors of CKD [16, 47–50], we hypothesize that CMI correlates with reduced eGFR and is capable of being a premier indicator of eGFR decrement. In our present study, regression analyses revealed the intensive and direct associations of CMI with eGFR value and eGFR reduction in both genders, confirming above hypothesis. Moreover, we observed a breakpoint in the association between CMI and eGFR value, which meant the change of eGFR shifted from decrease to increase after CMI reached certain value. And this phenomenon was robust and persistent across genders. Although the exact mechanism underlying this paradoxical phenomenon is still unclear, some previous findings can also give us a clue for possible explanations. While obesity was commonly considered to associate with the decline of eGFR [8, 15], studies also found a protective effect of obesity on CKD patients [51, 52], and this protective effect could be explained by the evidence that excessive adipose tissue might temper the deleterious effects of inflammatory factors by sequestering them [53, 54]. Consistent with this theory, the breakpoint was located at high levels of CMI (> 75 percentile) in each gender, which meant that the protective effect became more predominant than the destructive effect only when fatty tissue accumulated to a sufficient volume. Another possible explanation was the residual confounders. Although we had adjusted several factors that related to CMI and eGFR, there were still some confounders that we did not take into our regression equations. Therefore, some uncovered confounding factors could influence our results and caused this paradox. In order to validate the paradox founded in the present study, studies with prospective design and consideration of more modifiers are needed in the future.

Further, we confirmed the association between CMI and reduced eGFR was positive and strong. We found that the risk of reduced eGFR was proportionally increased across the quartiles of CMI, which was different from the breakpoint phenomenon in the association between CMI and eGFR value. Given that the breakpoint of CMI located behind the 75 percentile in both genders, we could easily figure out that the limited increase of eGFR in CMI levels above the breakpoint has minimal effect on the whole inverse trend of CMI with reduced kidney function (eGFR< 60 ml/min per 1.73m^2^). We also noticed that males had a slightly higher risk of developing reduced eGFR when compared with their female counterparts. But the interaction test revealed that this disadvantage was statistically insignificant. However, although we could not conclude that males were under greater risk of reduced eGFR, we still elucidated that CMI had a robust association with eGFR reduction in both genders, identifying the utility and stability of CMI for the risk stratification of reduced eGFR.

Findings from ROC analyses provided compelling evidence for CMI to be a screening marker of CKD. In males, CMI possessed greatest AUC value, although it did not have a significant larger discriminating ability than VAI, the simple and generalized equation for both sexes would be a vantage. With a sensitivity of 88.9%, CMI demonstrated its capability to function well in the screening test, since more suspicious patients should be positive in this kind of examination. It was worthy to note that neither of CMI, VAI and LAP had a significant greater AUC than WC, which was beyond our expectation, this could be explained by the lacking of sample size since the absolute number and prevalence of reduced eGFR were much lower in males than in females. Further studies are needed to validate this intriguing finding. In females, CMI did an even better job as it performed better than other indexes significantly except VAI. Although exhibited a negligible disadvantage in discriminating eGFR reduction, CMI showed a much higher sensitivity than VAI, thus the applicative value for CMI was still superior than VAI among females. In conclusion, with great discriminative ability and high sensitivity value, CMI showed its potential for becoming an economic screening marker to filter out people with reduced eGFR.

Mounting evidence has emerged to reveal the mechanism under the association between CMI with CKD. First, mechanism of dyslipidemia in CKD has already been elucidated. Hypertriglyceridemia is a multifactorial phenomenon, and is partially due to diminished catabolism. This reduction is an outcome of depressed lipoprotein lipase (LPL) activity, which is responsible for the hydrolysis of TG [55]. And the decreased LPL activity has been identified to be caused by secondary hyperparathyroidism induced insulin resistance (IR) and excess of lipase inhibitors like apolipoprotein (Apo) C-III in the progression of CKD [56, 57]. For another aspect, hepatic Apo A-I gene expression and hepatic lecithin cholesterol acyl transferase (LCAT) mRNA expression are downregulated during the course of CKD, and then this change attributes to a lower plasma Apo A-I level and decreased LCAT activity [58, 59]. Since Apo A-I is an essential functional component of HDL-C [60], the concentration of HDL-C in blood is consequently decreased. Similarly, the normal function of HDL-C is also impaired because of inadequate LCAT activity, which is important in the process of transporting cholesterol to HDL in peripheral tissues [60]. Second, basic research has also revealed the intrinsic mechanism between obesity with CKD. Adipocytes produce a series of factors, such as angiotensinogen, precursor of angiotensin II and leptin, which may trigger glomerular hypertension through local renin-angiotensin-aldosterone system (RAAS) or sympathetic system, then leading to the decrease of eGFR [8]. Furthermore, inflammation involves in the association between obesity with CKD as well, macrophages are found to accumulate in the kidney of obese animals in the effect of angiotensin [61]. Some inflammatory cytokines such as interleukin-6 (IL-6) and Tumor necrosis factor-α (TNF-α) have also been implicated in obesity-related CKD [62]. Therefore, obesity could promote the progression of renal disease through several, partially overlapping mechanisms. Sum up above two points, as composites of CMI, dyslipidemia and obesity have experiment confirmed relationship with CKD. Thus, the association between CMI with reduced eGFR observed in our study has a concrete foundation from basic researches.

There are still some limitations in our study that needed to be mentioned. First, due to the cross-sectional design, our work can only provide evidence about the strong association between CMI and reduced eGFR, but information about the causality of this relationship need further prospective studies to confirm. Second, it has an inherent limitation of not being a randomized study. Although multivariable adjustments were performed for potential confounding factors, a possible effect of unmeasured variables such as C-reactive protein cannot be excluded. Third, our study participants were recruited from rural area of northeast of China, whether the results can be applied to populations of different areas or races require more studies to investigate. Finally, our diagnosis based on a single biochemistry test, the creatinine value could be influenced by uncertain factors, thus the accuracy of eGFR could be mildly disturbed.

## Conclusions

In summary, our work was the first study to reveal the relationship between CMI and reduced eGFR which was independent of prevalent CVD, medication used, and conventional cardiovascular risk factors. Thus, these data provide strong evidence of a unique, independent and economic role of CMI in a high burden of kidney disease. These findings have important implications for guiding primordial prevention and understanding the mechanisms underlying VAT-mediated renal injury.

## Abbreviations

AUC: Area under the curve
BMI: Body mass index
CI: Confidence internal
CKD: Chronic kidney disease
CMI: Cardiometabolic index
CVD: Cardiovascular disease
DBP: Diastolic blood pressure
DM: Diabetes mellitus
eGFR: Estimated glomerular filtration index
ESRD: End stage renal disease
FPG: Fasting plasma glucose
HDL-C: High density lipoprotein cholesterol
IL: Interleukin
IR: Insulin resistance
LAP: Lipid accumulation product
LCAT: Lecithin cholesterol acyl transferase
LPL: Lipoprotein lipase
NCRCHS: Northeast China Rural Cardiovascular Health Study
NO: Nitric oxide
ORs: Odds ratios
RAAS: Renin-angiotensin-aldosterone system
ROC: Receiver operating characteristic curve
SAT: Subcutaneous adipose tissue
SBP: Systolic blood pressure
SD: Standard deviation
TG: Triglyceride
TG/HDL-C: Triglyceride to high density lipoprotein cholesterol ratio
TNF-α: Tumor necrosis factor alpha
VAI: Visceral adiposity index
VAT: Visceral adipose tissue
WC: Waist circumference
WHtR: Waist to height ratio
